# Study on the potential diagnostic value of metabolomics changes in different biological fluids for aspiration pneumonia

**DOI:** 10.1186/s12890-025-03519-x

**Published:** 2025-02-04

**Authors:** Lianghui Chen, Yazhen Chen, Fansen Lin, Jianbao Wang, Hongzhi Gao, Yuqi Liu

**Affiliations:** 1 Department of Critical Care Medicine, Second Affiliated Hospital of Fujian Medical University, Quanzhou, 362000 People’s Republic of China; 2 Department of Neurosurgery, Second Affiliated Hospital of Fujian Medical University, Quanzhou, 362000 People’s Republic of China

**Keywords:** Aspiration pneumonia, Metabolic profiles, Biomarker, Liquid chromatography with tandem mass spectrometry

## Abstract

**Background:**

Aspiration pneumonia (AP) is a type of lung inflammation caused by the aspiration of food, oropharyngeal secretions, or gastric contents. This condition is particularly common in older adults and individuals with impaired swallowing or consciousness. While the diagnosis of AP relies on clinical history, swallowing assessments, and imaging, these methods have significant limitations, often leading to underdiagnosis or misdiagnosis. Reliable biomarkers for AP diagnosis are lacking, making early detection and treatment challenging.

**Methods:**

Nineteen patients diagnosed with pneumonia were included in this study, divided into two groups: AP (*n* = 10) and non-AP (*n* = 9). Biological fluid samples, including bronchoalveolar lavage fluid (BALF), saliva, serum, sputum, and urine, were analyzed using non-targeted liquid chromatography with tandem mass spectrometry (LC-MS/MS). Differential metabolites were identified using fold change analysis, statistical significance, and receiver operating characteristic (ROC) curve analysis to evaluate their diagnostic potential. Spearman correlation was used to examine the relationship between selected metabolites and clinical parameters.

**Results:**

Significant metabolic differences were found between AP and non-AP patients, with many different metabolites identified across biological fluids. Dehydroepiandrosterone sulfate (DHEAS), Androstenediol-3-sulfate (ADIOLS), and beta-muricholic acid were identified as key biomarkers through fold change analysis and ROC curve analysis, showing consistent increasing or decreasing trends in BALF, sputum, and serum samples. DHEAS was found to be negatively correlated with the Acute Physiology and Chronic Health Evaluation II (APACHE II) (*r* = − 0.619, *p* = 0.005) in BALF sample. The area under curve (AUC) values showed that these molecules could serve as effective biomarkers for AP.

**Conclusions:**

This study identifies DHEAS, ADIOLS and beta-muricholic acid as promising biomarkers for AP, with the potential to improve early diagnosis and treatment. These findings underscore the clinical value of metabolomics in developing diagnostic tools for AP, facilitating better clinical management and patient outcomes. Further research is required to validate these biomarkers in larger cohorts and explore their mechanistic roles in AP pathophysiology.

**Supplementary Information:**

The online version contains supplementary material available at 10.1186/s12890-025-03519-x.

## Background

Aspiration pneumonia (AP) is a form of lung inflammation caused by the aspiration of food, oropharyngeal secretions, or gastric contents. The incidence of AP in patients with community-acquired pneumonia ranges from 5 to 15% [1]. AP is particularly prevalent in older adults and individuals with impaired consciousness, swallowing dysfunction, or a weakened cough reflex. The clinical manifestations of AP range from no symptoms to severe distress with respiratory failure [2], complicating diagnosis.

Risk factors for AP include consciousness impairment, impaired swallowing, impaired cough reflex, and increased gastric content reflux. Based on clinical symptoms and aspiration history, AP can be categorized into overt AP, which presents with symptoms such as irritating cough, shortness of breath, or severe distress immediately after aspiration, and concealed AP, which often manifests without acute symptoms, leading to chronic inflammation, weight loss, and malnutrition over time.

Current diagnostic methods for AP include clinical history, swallowing assessments (such as water swallow tests, videofluoroscopic swallowing studies (VFSS), and flexible endoscopic evaluation of swallowing (FEES)), and chest imaging [2, 3]. While these methods are commonly used, they are often limited in identifying concealed AP, a subtype where aspiration occurs intermittently or without obvious symptoms, leading to underdiagnosis. Moreover, reliance on clinical history is subjective, and diagnostic imaging can overlap with other types of pneumonia, further complicating accurate identification. In a cohort of 59 pathology-confirmed cases of AP, only 4 cases (6.8%) were suspected of aspiration before death, highlighting that 93.2% of AP cases may go undiagnosed [4]. The prognosis of patients with AP can be significantly improved with prompt diagnosis and treatment.

An ideal biomarker for AP would enable early detection, even in the absence of a clinical history, and reduce the need for invasive diagnostic procedures. Several studies have investigated alpha-amylase as a potential biomarker for AP [5, 6]. Bronchoalveolar lavage fluid (BALF) alpha-amylase concentrations have been linked to traditional aspiration risk factors; however, the relevance of these findings to AP diagnosis remains unclear. Metabolomics has emerged as a powerful tool for identifying biomarkers that could aid in the diagnosis of AP. Recent studies have highlighted the potential of metabolomics-based diagnostic biomarkers to improve the diagnosis of pneumonia [7, 8]. However, to date, no study has comprehensively investigated the metabolic differences across multiple biological fluids in patients with AP.

This study aims to address this gap by employing a non-targeted metabolomics approach to identify and compare the metabolic profiles in BALF, saliva, serum, sputum, and urine from AP patients. The results of this study could lead to the development of biomarkers for early diagnosis, thereby overcoming the current diagnostic challenges associated with AP.

## Methods

### Study design

Samples were collected from patients admitted to the respiratory medicine, intensive care unit (ICU), and neurology departments of four hospitals in China between January and December 2022. The study was approved by the Ethics Committee of Scientific Research Review at the Second Affiliated Hospital of Fujian Medical University (No. 2022 −500), and all procedures were conducted in accordance with the relevant ethical guidelines and regulations. Written informed consent was obtained from all participants. Clinical trial number: not applicable.

A total of 19 patients diagnosed with pneumonia [9, 10] were included in the study and divided into two groups: the AP group (*n* = 10) and the non-AP group (*n* = 9). The diagnostic criteria for AP included a history of witnessed macroaspiration, risk factors for aspiration, and a gravity-dependent distribution of shadows on chest imaging [2]. Additionally, aspiration must be confirmed either during bronchoscopy or throughout hospitalization [11]. Patients with conditions such as parkinsonism, stroke, tracheotomy, or myasthenia gravis were considered at risk for aspiration. The non-AP group included patients diagnosed with pneumonia who had no history or clinical evidence of aspiration or related risk factors.

Exclusion criteria included evidence of airway bleeding, pulmonary tuberculosis, malignancy, or pulmonary embolism. Patients who were unable to undergo bronchoalveolar lavage for other reasons were also excluded.

### Sample collection and preparation

With patient consent, sputum and BALF were collected under bronchoscopy. The lesion location was identified using chest imaging, and the most significant site was selected for bronchoalveolar lavage. Additionally, saliva, peripheral venous blood, and urine samples were collected. Blood samples were incubated for 30 min, followed by centrifugation at 1500×g for 10 min. Urine samples were similarly centrifuged at the same speed. All collected samples—BALF, saliva, sputum, serum, and urine supernatant—were stored at − 80 °C until further use.

Upon analysis, samples were thawed on ice, and aliquots were mixed to prepare quality control (QC) samples. A blank sample was also prepared by adding water in place of biofluid. For metabolite extraction, 300 µL of extraction reagent (methanol containing mixed isotope internal standards, including citric acid d4, DNOP-d4, L-tryptophan-d5, and N-benzoyl-D-phenylalanine) was added to 100 µL of the sample. The mixture was shaken at 1000 rpm and vortexed for 10 min. Following vortexing, the samples were centrifuged at 15,000 rpm at 4 °C for 10 min. The supernatant was transferred to a protein precipitation plate for filtration. After filtration, an equal volume of filtrate was transferred to a new centrifuge tube and freeze-dried. When ready for analysis, the lyophilized sample was reconstituted with 200 µL of reconstitution solvent (50% methanol), and 10 µL was loaded onto the mass spectrometer for quantitative analysis.

### LC-MS/MS analysis

Materials: Ammonium formate (Sigma-Aldrich), Methanol, Optima™ LC/MS Grade (Fisher Scientific), Acetonitrile, Optima™ LC/MS Grade, (Fisher Scientific), Formic acid, LC/MS Grade, (Fisher Scientific), ddH2O (water from Merck MilliporeTM water purification system).

Metabolic extracts were analyzed using a Thermo Scientific™ Vanquish™ UHPLC coupled with Q-Exactive™ PLUS mass spectrometer (ThermoFisher, USA) under data-dependent acquisition (DDA) mode. The metabolites were separated on BEH C18 column (Waters, 100 mm × 2.1 mm, 1.7 μm) using an 18-minute gradient with organic phase increased from 2 to 100% at 40 °C in 12 min (solvent A: 0.1% formic acid, 2 mM ammonium formate in water; solvent B: 0.1% formic acid, 2 mM ammonium formate in methanol). The metabolites were also separated on BEH AMIDE column (Waters Co., USA, 1.7 μm, 2.1 × 100 mm) using a 12-minute gradient with the organic phase increased from 2 to 98% in 10 min at 50 °C (solvent A: water, 25 mM ammonium formate, pH 9.0, solvent B: acetonitrile). The MS detection was carried out in positive and negative ion modes, respectively. A full survey scan was obtained at a 70,000 resolution for the m/z range of 70 to 1050 and followed by 10 MS/MS scans in HCD mode at a 17,500 resolution (Supplementary Methods, Additional File 1).

### Metabolite identification and quantification

DDA raw data including biofluids, QC and blank samples was extracted, peak features-identified using Compound Discoverer v 3.2 software (Themo). Compounds were identified by comparison to library entries of purified standards or public MS/MS entities. The library based on authenticated standards that contains the retention time (RT), mass to charge ratio (m/z), and chromatographic data (including MS/MS spectral data) for all molecules present in the library with mzValt 2.3. Other public libraries such as NIST2022, mzCloud 2021B and mzCloud 2023 were used for metabolites identification. Furthermore, biochemical identifications as level 1 are based on three criteria: RT within a narrow window of the proposed identification, accurate mass match to the library–/- 10ppm, and the MS/MS scores between the experimental data and authentic standards, filtered greater than 0.7. Biochemical identifications as level 2 are based on three criteria: accurate mass match to the library–/- 10ppm, and the MS/MS scores greater than 0.7.

### Statistical analysis

Metabolite intensities were exported from Compound Discoverer 3.2. Metabolites with more than 20% missing values were excluded from the analysis, and missing data were imputed using the K-Nearest Neighbors (KNN) method. Metabolite intensities were normalized using variance stabilizing normalization (VSN). The relative standard deviation (RSD) of metabolite intensities within QC samples was calculated using the numpy package (v.1.21.0) in Python. Metabolites with an RSD greater than 0.3 or identified as xenobiotics were excluded from further analysis.

Partial least squares discriminant analysis (PLS-DA) was performed using the ropls function in R (v.4.1.0). Differential metabolites were identified using an unpaired two-tailed Student’s t-test. A *p*-value < 0.05 were considered significant [12, 13]. Metabolite enrichment analysis was conducted using the Kyoto encyclopedia gene and genome database (KEGG) database. All detected metabolites were used as the background, and enrichment was calculated using the Fisher’s exact test (one-sided) with pathway class annotations. Pathways with adjusted *p*-values (Benjamini-Hochberg method) less than 0.05 were considered significant. The direction of change for each pathway was determined by calculating the median fold change values of significant metabolites in the pathway.

Analysis of Clinical Information: For continuous variables, data were expressed as the mean ± standard deviation (SD) if normally distributed, or as the median with interquartile range (IQR) if not normally distributed. Categorical data were presented as frequencies and percentages. Group comparisons were performed using the Student’s t-test or Mann-Whitney U test for quantitative variables, and Fisher’s exact test for categorical variables. Missing values were imputed using multiple imputation (Table S1, Additional File 1).

Receiver operating characteristic (ROC) curves were used to evaluate the diagnostic ability of selected differential metabolites for AP. Spearman’s rank correlation was used to assess the relationship between differential metabolites and clinical parameters among patients with AP. Data analysis was performed using GraphPad Prism (v.8.0), and SPSS Statistics (v.27.0). A two-sided *α* of less than 0.05 was considered statistically significant.

**Fig. 1:**
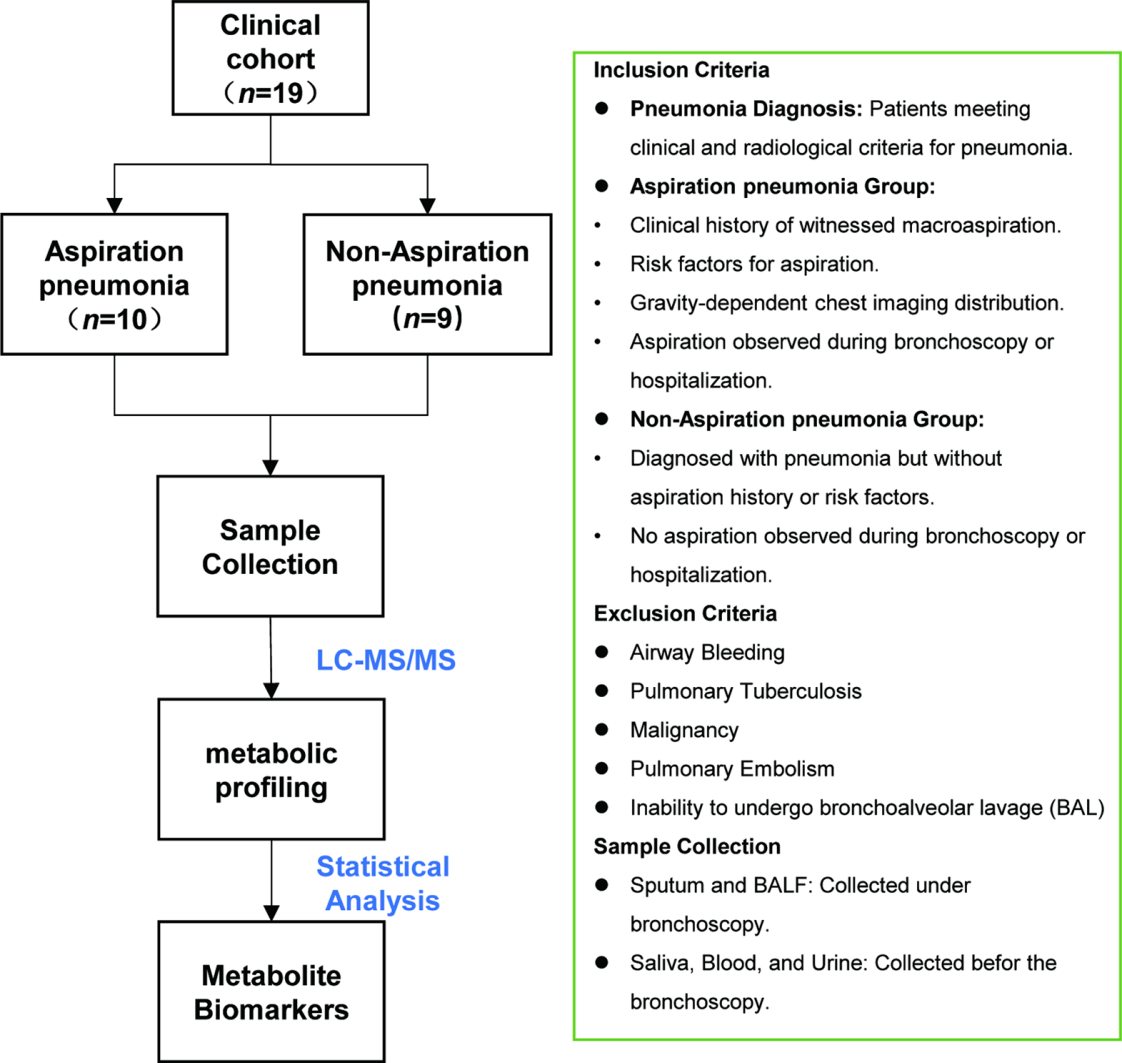
The schematic diagram of this study

The schematic diagram of this study was presented in Fig. 1.

## Results

### Baseline data

Nineteen patients diagnosed with pneumonia were included in the study and subsequently divided into two groups: the AP group (*n* = 10) and the non-AP group (*n* = 9). As shown in Table 1, there were no significant differences in age, sex, or smoking history between the groups (*p* > 0.05). However, the proportion of neurological diseases was significantly higher in patients with AP compared to those without AP (*p* < 0.05). Additionally, AP patients had significantly higher Acute Physiology and Chronic Health Evaluation II (APACHE II) scores (*p* < 0.05), reflecting greater disease severity. During hospitalization, sedatives and corticosteroids were used more frequently in the AP group than in the non-AP group (*p* < 0.05).

**Table 1:** Demographic and clinical characteristics of the 19 subjects enrolled in this study

Characteristic	Non-AP(*n* = 9)	AP(*n* = 10)	*p* value
Age(years)	55.89 ± 12.00	61.80 ± 10.68	0.272
Sex, male	7(77.8)	9(90)	0.582
Smoking history	3(33.3)	1(10.0)	0.303
Underlying diseases			
Respiratory diseases	5(55.6)	3(30)	0.370
Cardiovascular diseases	4(44.4)	6(60.0)	0.656
Liver diseases	2(22.2)	1(10.0)	0.582
Neurological diseases	1(11.1)	7(70.0)	0.020
Diabetes	3(33.3)	3(30.0)	1.000
WBC (×10^9^/L)	11.11 ± 3.92	15.19 ± 9.32	0.229
PCT (ng/ml)	1.67(0.35–7.03)	1.01(0.31–8.58)	0.661
Cholesterol (mmol/L)	3.05 ± 0.73	2.70 ± 1.31	0.476
Albumin (g/L)	33.85 ± 5.41	33.87 ± 4.57	0.995
D-BIL (µmol/L)	7.10(4.11–9.20)	5.20(3.17–6.70)	0.278
I-BIL (µmol/L)	2.41(1.41–13.87)	6.80(4.00-11.13)	0.243
Lac (mmol/L)	1.14 ± 0.28	1.34 ± 0.67	0.417
APACHE II score	7.00(7.00-9.50)	18.00(12.00-22.25)	< 0.001
Drug treatment			
Corticosteroids	3(33.3)	10(100)	0.003
Sedative	9(100)	4(40)	0.011
Invasive ventilation	5(55.6)	9(90.0)	0.141
Shock	3(33.3)	0(0)	0.087
Hospital LOS (days)	17.00(14.00-28.50)	26.50(19.00–71.00)	0.113
ICU admission	6(66.7)	9(90)	0.303

Values are mean ± SD, n (%), or median (interquartile range)

AP: aspiration pneumonia; WBC: white blood cell; PCT: procalcitonin; D-BIL: direct bilirubin; I-BIL: indirect bilirubin; Lac: lactic acid; APACHE II: Acute Physiology and Chronic Health Evaluation II; LOS: length of stay; ICU: intensive care unit

### Global metabolic profiling

**Fig. 2:**
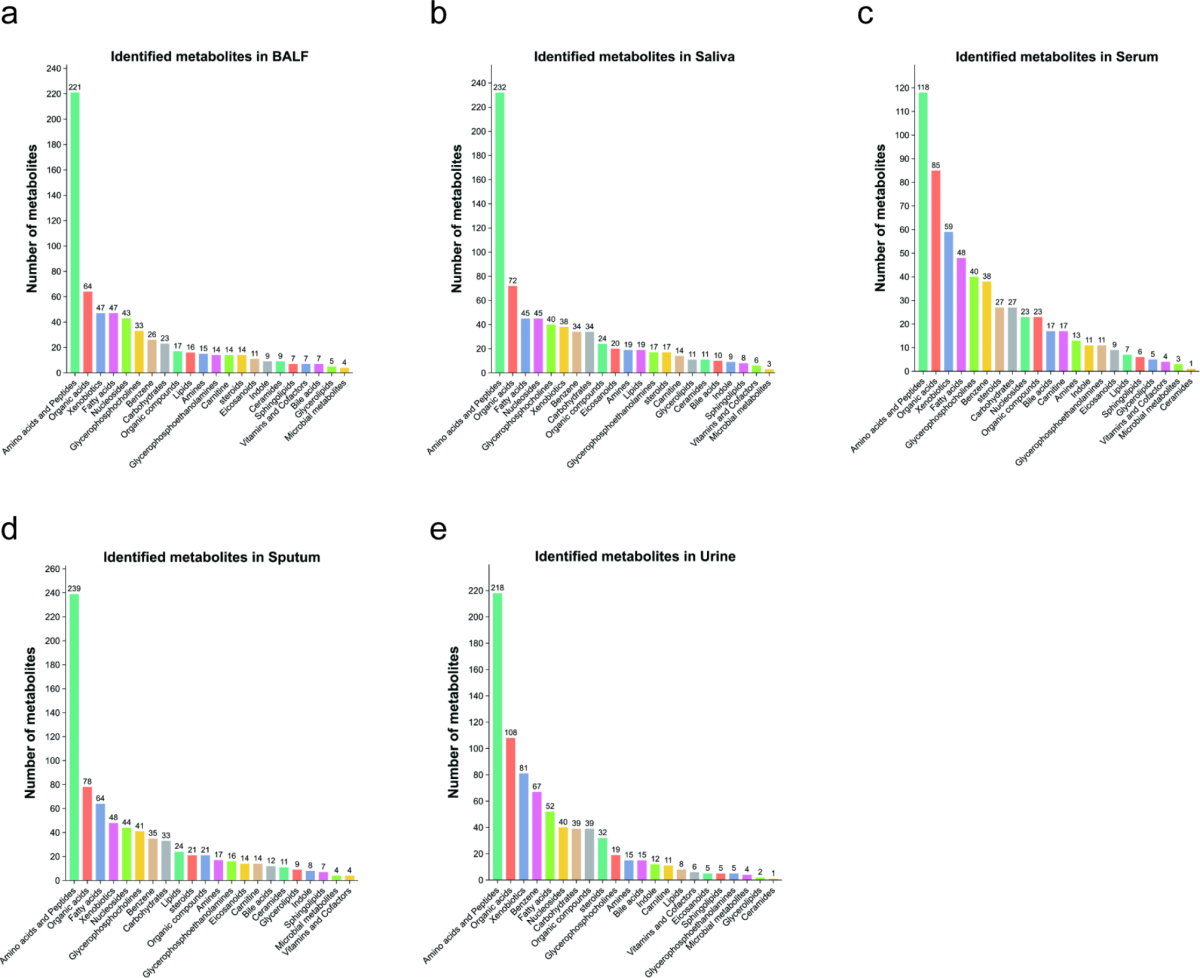
Metabolite category statistics. (**a-e**) Classification of metabolites identified in bronchoalveolar lavage fluid (BALF), saliva, serum, sputum, and urine samples. The metabolites are grouped based on their chemical properties, with amino acids and peptides, organic acids, and fatty acids being the most abundant categories

Non-targeted liquid chromatography with tandem mass spectrometry (LC-MS/MS) analysis identified 653 molecules in BALF, 726 in saliva, 592 in serum, 764 in sputum, and 784 in urine samples (Fig. 2). Amino acids and peptides were the most abundant metabolite classes across BALF, saliva, serum, and sputum, followed by organic acids and fatty acids. However, the metabolic composition of urine was distinct, with benzene ranking third in abundance, reflecting the unique profile of this biological fluid. The complete information of metabolites identified in different sample types were shown in Table S2-S6, Additional File 2.

**Fig. 3:**
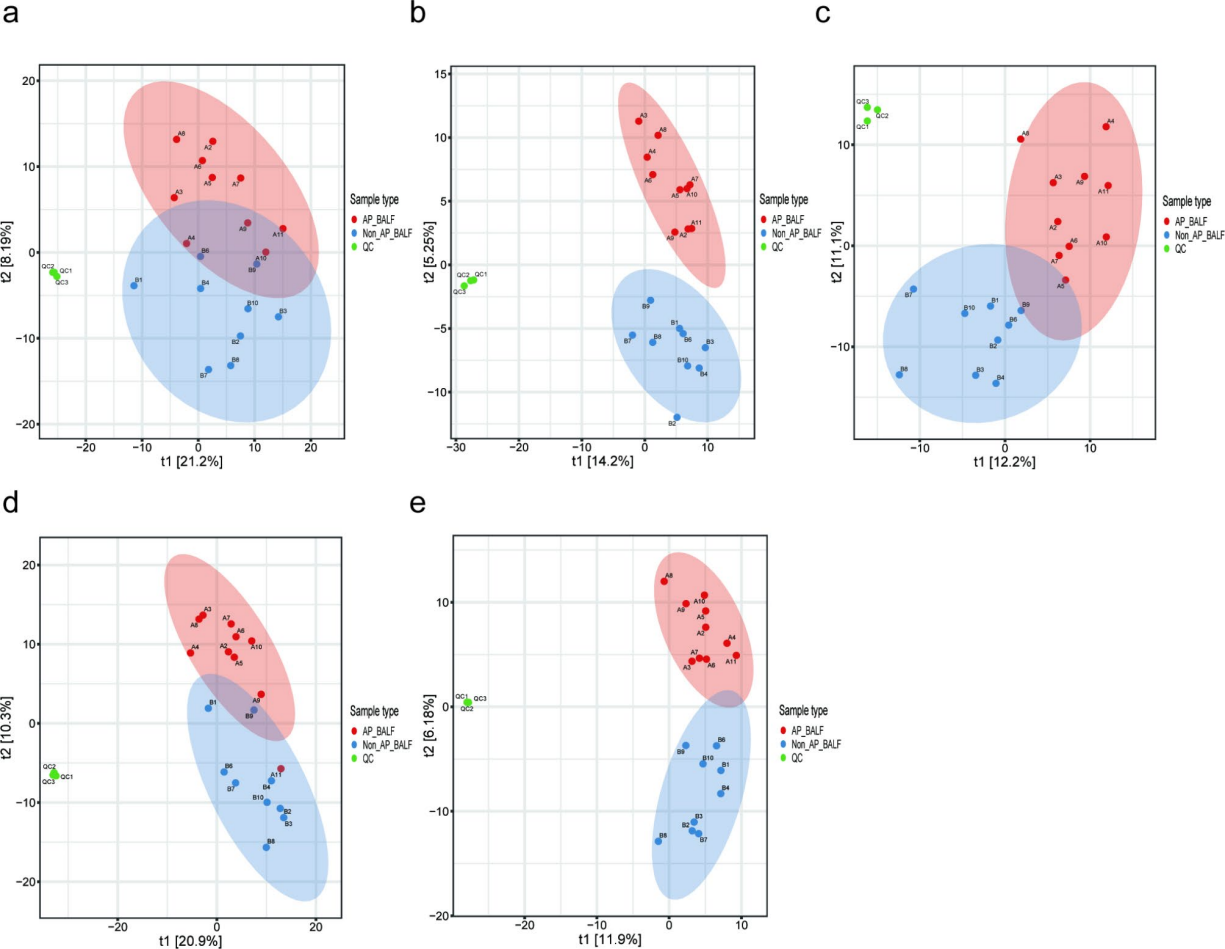
Partial least squares discriminant analysis (PLS-DA) of metabolomic profiles from five sample types. (**a-e**) PLS-DA was used to assess the metabolic differences between aspiration pneumonia (AP) and non-aspiration pneumonia (non-AP) using bronchoalveolar lavage fluid (BALF), saliva, serum, sputum, and urine samples. Quality control (QC) samples were included to assess the reliability of the data. Each point represents an individual sample, and the analysis shows how AP and Non-AP groups are distinguished based on their metabolic profiles

Xenobiotics were excluded from further analysis due to their limited relevance. PLS-DA revealed clear differences in metabolic profiles between patients with AP and those without AP. QC samples were tightly clustered in the PLS-DA plot, indicating excellent analytical repeatability and instrument stability (Fig. 3). Only metabolites with RSD below 30% were included in subsequent analyses to ensure the reliability of the results (Fig. S1, Additional File 1).

### Metabolic alteration between patients with AP and patients with non-AP

**Fig. 4:**
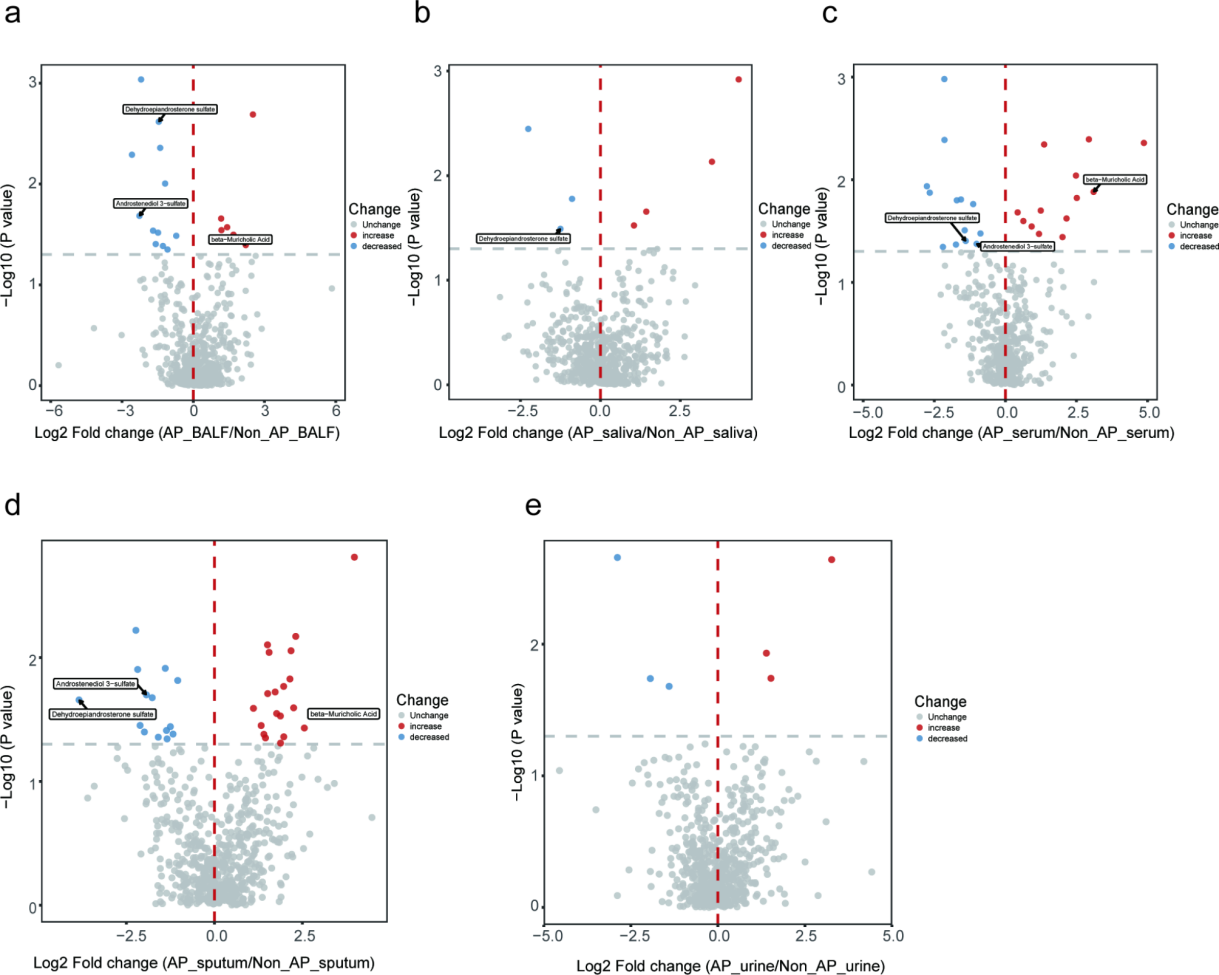
Volcano plot of differential metabolites. (**a**) Differential analysis of metabolites in BALF samples. The X-axis represents the log2-transformed fold change, and the Y-axis represents the P-value based on log10-transformed values. Metabolites with significant differences are located in the upper left and upper right quadrants. The metabolites that decreased are marked in blue, the metabolites that increased are marked in red, and metabolites with no significant difference are shown in gray. (**b-e**) Differential analysis of metabolites in saliva, serum, sputum, and urine samples

**Fig. 5:**
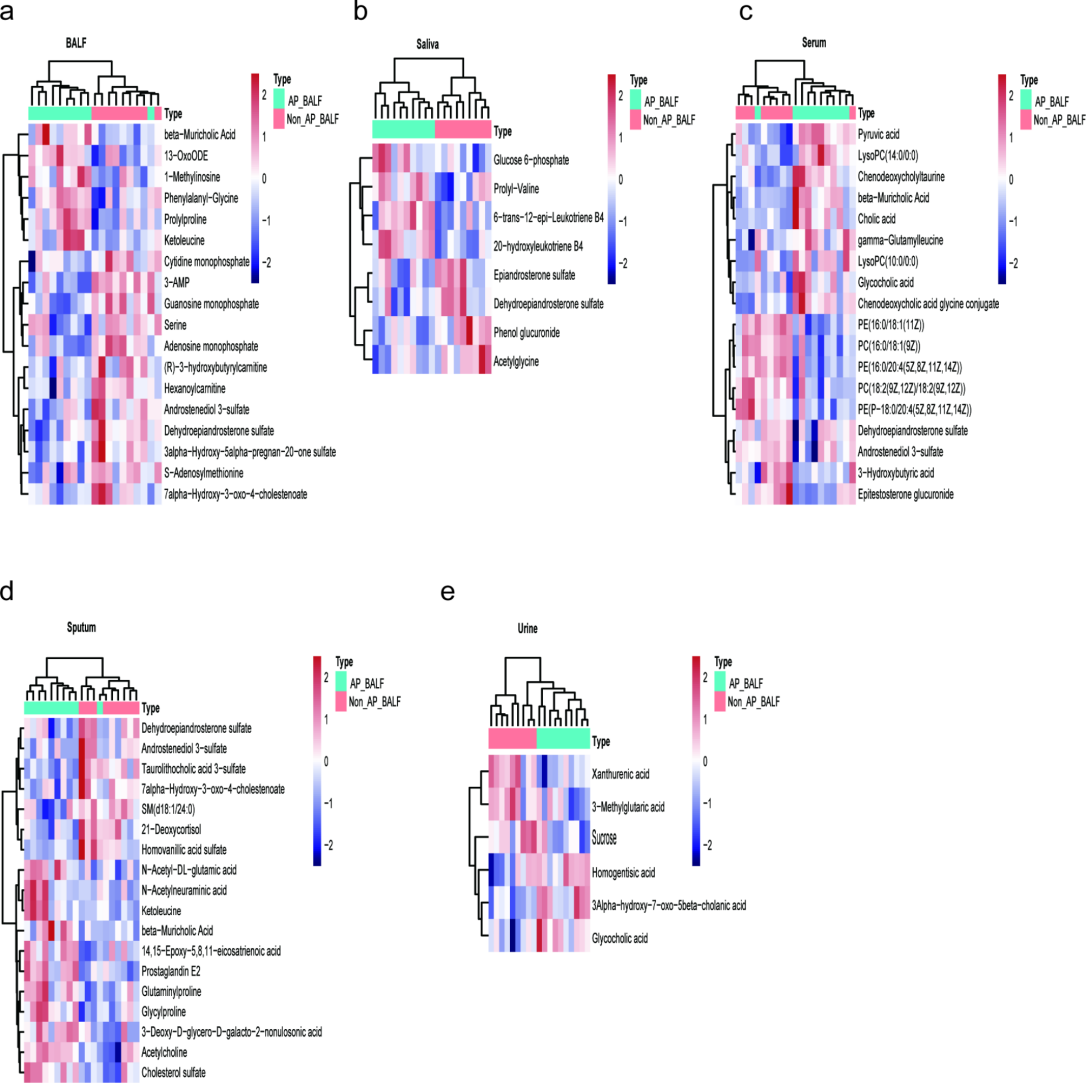
The abundance of metabolites with significant differences in different samples. (**a**) The abundance of metabolites with significant differences in BALF samples. Each row represents a metabolite, and each column represents a sample. The color of each cell indicates the abundance of the corresponding metabolite in the respective sample. Changes in cell color reflect alterations in metabolite abundance. (**b-e**) The abundance of metabolites with significant differences in saliva, serum, sputum, and urine samples

Differential metabolites between patients with AP and patients with non-AP were identified using a *p*-value of < 0.05. In BALF samples, 18 metabolites were significantly altered, with 6 showing increased levels and 12 showing decreased levels in AP patients compared to non-AP patients (Fig. 4a). In saliva samples, 8 metabolites were significantly different, with 4 showing increased levels and 4 showing decreased levels (Fig. 4b). Serum samples contained 27 significantly altered metabolites, of which 14 were increased and 13 were decreased (Fig. 4c). In sputum samples, 33 metabolites were significantly different, with 19 showing increased levels and 14 showing decreased levels (Fig. 4d). Lastly, 6 metabolites in urine samples were significantly altered, with 3 showing increased levels and 3 showing decreased levels (Fig. 4e). Heatmaps illustrating the relative abundance of the most representative metabolites for each sample type are presented in Fig. 5, with a full list of differential metabolites provided in Additional File 3 (Tables S7-S11).

Dehydroepiandrosterone sulfate (DHEAS), androstenediol-3-sulfate (ADIOLS), and beta-muricholic acid demonstrated consistent trends across BALF, sputum, and serum samples (Fig. S2, Additional File 1), with DHEAS and ADIOLS showing decreased levels in AP patients and beta-muricholic acid showing increased levels. These alterations highlight their potential as reliable biomarkers for AP diagnosis.

BALF, sputum, and serum are critical in pneumonia research due to their close connection to lung physiology and immune responses. BALF reflects local inflammatory and immune activity in the lower respiratory tract, providing insights into lung-specific metabolic changes. Sputum, which represents secretions from both the upper and lower airways, captures a wider range of respiratory tract alterations. Serum, as a systemic biofluid, offers an overview of the body’s inflammatory and metabolic responses to pneumonia. Together, these fluids provide a comprehensive perspective that enhances biomarker discovery and deepens understanding of pneumonia pathophysiology.

### Pathway enrichment analysis

**Fig. 6:**
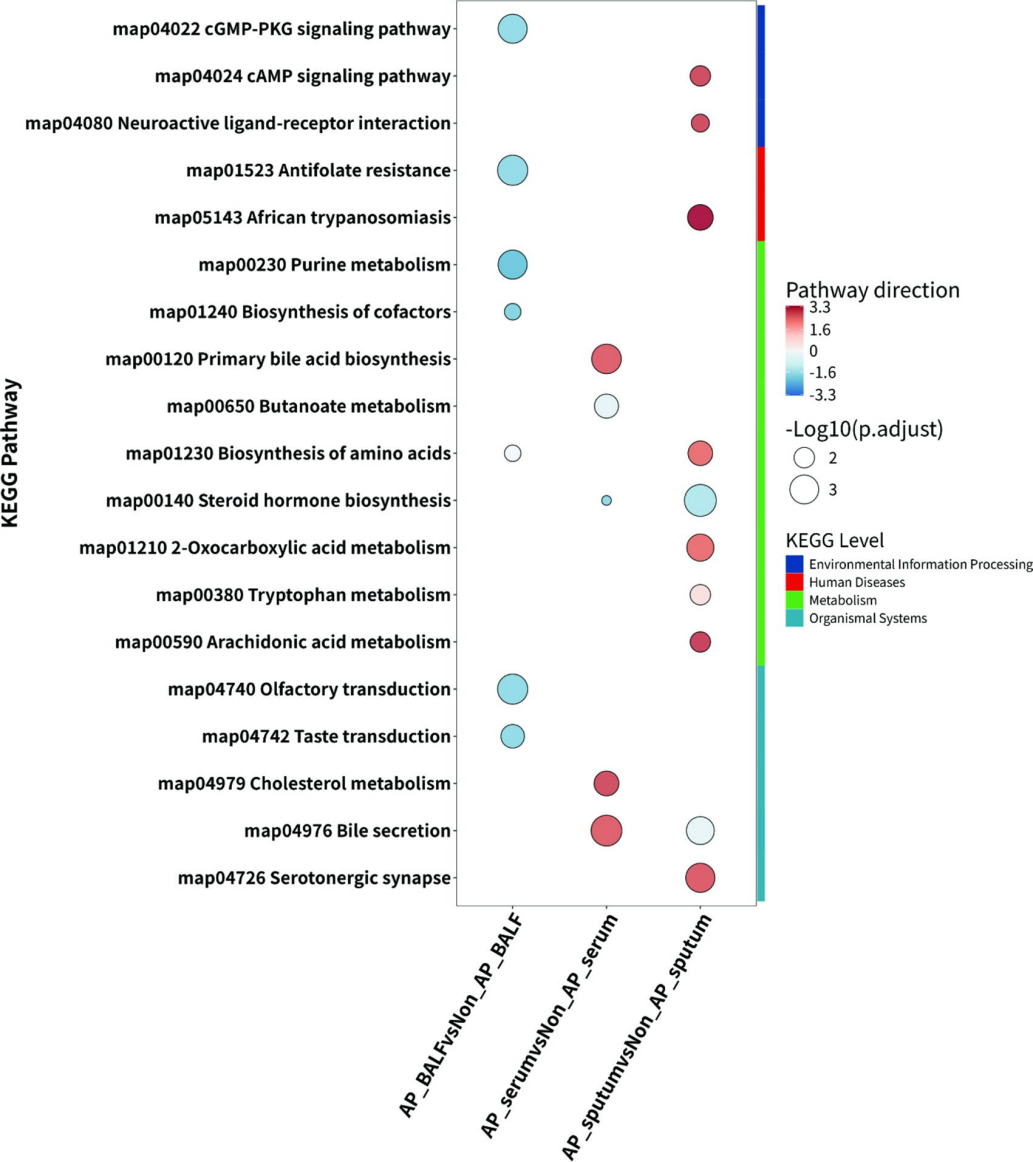
KEGG pathway enrichment analysis for each sample type. The ordinate represents the enriched KEGG pathways, and the abscissa represents the sample types. The size of the bubble indicates the significance of the enriched KEGG pathway. The color of the bubble reflects the regulatory direction of the pathway, with blue representing downregulation and brown representing upregulation. The color depth indicates the intensity of the regulation. The colors along the ordinate represent the first-order classification of the enriched KEGG pathways

KEGG pathway enrichment analysis identified several dysregulated pathways associated with AP across the various biological fluids. In BALF samples, enriched pathways included olfactory transduction, cGMP − PKG signaling, purine metabolism, and amino acid biosynthesis, among others (Fig. S3a). In serum samples, dysregulated pathways included cholesterol metabolism, primary bile acid biosynthesis, and bile secretion, and so on (Fig. S3b). Sputum samples showed significant enrichment in pathways such as arachidonic acid metabolism, steroid hormone biosynthesis, and bile secretion, and other pathways (Fig. S3c). All enriched pathways for different types of samples are displayed in Fig. 6. Importantly, bile secretion pathways were significantly enriched in both serum and sputum samples. Previous research has emphasized the role of bile acids as signaling molecules in immune system regulation [14]. A complete list of these pathways was shown in Table S12-S15, Additional File 4.

### Identification of biomarkers

**Fig. 7:**
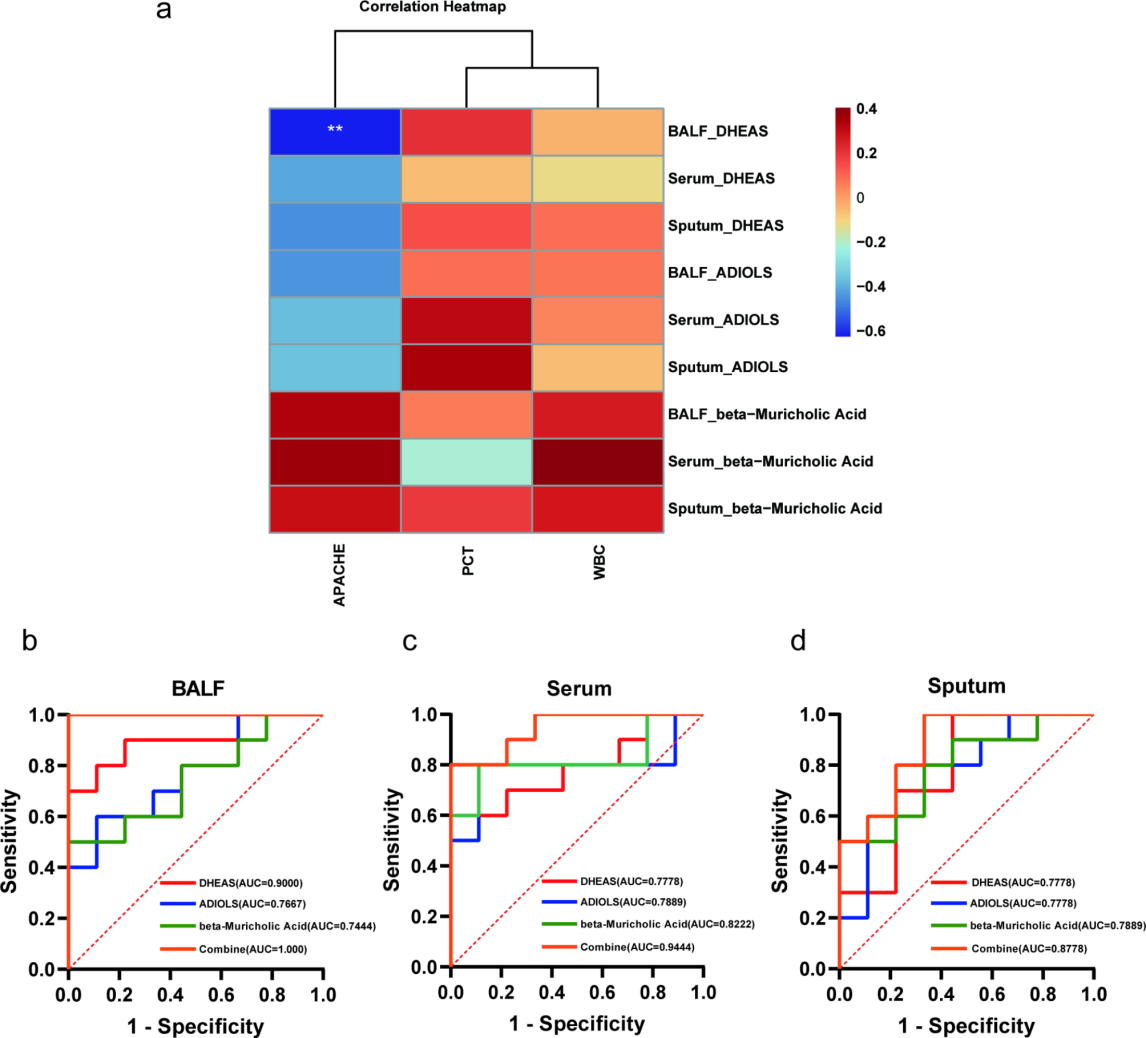
Diagnostic performance and clinical correlation of selected differential metabolites. (**a**) Clustering correlation heatmap of selected differential metabolites. Each row represents a selected differential metabolite, and each column represents a clinical parameter. The color of each cell represents the correlation between the selected metabolite and the clinical parameter. Brown indicates a positive correlation, blue indicates a negative correlation, and the color depth reflects the intensity of the correlation (* *P* < 0.05). (**b-d**) ROC curve analysis of selected differential metabolites

The diagnostic potential of DHEAS, ADIOLS, and beta-muricholic acid was validated through ROC curve analysis. In BALF, DHEAS demonstrated excellent diagnostic performance with an area under curve (AUC) of 0.9000, and its levels were negatively correlated with APACHE II scores (*r* = -0.619, *p* = 0.005, Spearman test), indicating an association with disease severity (Fig. 7a, Table S16, Additional File 5). ADIOLS exhibited moderate diagnostic performance in BALF (AUC = 0.7667), while beta-muricholic acid had an AUC of 0.7444 but lacked statistical significance (*p* = 0.0724). In serum samples, DHEAS, ADIOLS, and beta-muricholic acid displayed AUC values of 0.7778, 0.7889, and 0.8222, respectively, demonstrating moderate diagnostic potential. Similarly, in sputum samples, the AUC values for DHEAS, ADIOLS, and beta-muricholic acid were 0.7778, 0.7778, and 0.7889, respectively. The combined AUC values for these metabolites in BALF, serum, and sputum were 1.000, 0.9444, and 0.8778, respectively, further supporting their potential as diagnostic biomarkers for AP (Table 2; Fig. 7b-d).

**Table 2:** Diagnostic efficiency of common components among differential metabolites in BALF, sputum and serum

Metabolites	BALF			Serum			Sputum	
	AUC(95% CI)	*P*-value		AUC(95% CI)	*P*-value		AUC(95% CI)	*P*-value
DHEAS	0.9000(0.7547-1.000)	0.0033		0.7778(0.5650–0.9905)	0.0412		0.7778(0.5586–0.9970)	0.0412
ADIOLS	0.7667(0.5519–0.9815)	0.0500		0.7889(0.5595-1.000)	0.0338		0.7778(0.5607–0.9948)	0.0412
beta-Muricholic Acid	0.7444(0.5195–0.9693)	0.0724		0.8222(0.6160-1.000)	0.0179		0.7889(0.5825–0.9953)	0.0338
Combine	1.000(1.000–1.000)	0.0002		0.9444(0.8479-1.000)	0.0011		0.8778(0.7218-1.000)	0.0055

DHEAS: dehydroepiandrosterone sulfate; ADIOLS: androstenediol-3-sulfate; AUC: area under the curve; BALF: bronchopulmonary lavage fluid

## Discussion

In this study, we identified several metabolites, including DHEAS, ADIOLS, and beta-muricholic acid, as potential biomarkers for AP. These metabolites showed significant differences between AP and non-AP patients, and their diagnostic potential was further confirmed by ROC curve analysis. However, understanding the biological mechanisms through which these metabolites influence AP pathophysiology is crucial for interpreting their clinical relevance.

The concentration of DHEAS in BALF, sputum, and serum was significantly decreased in AP patients compared to non-AP patients, with levels in BALF negatively correlating with the APACHE II score. This suggests that DHEAS may reflect the severity of the disease. DHEAS is a steroid hormone primarily secreted by the adrenal gland [15], with dehydroepiandrosterone (DHEA) being its active form [16]. As a nutritional supplement, DHEA is widely used for its anti-inflammatory and immunomodulatory effects [17]. Previous studies have shown that DHEA enhances the integrity of the colon barrier by regulating oxidative stress and inflammation in colitis models [18]. Additionally, DHEA has been shown to inhibit the progression of nonalcoholic steatohepatitis by reducing lipid deposition, inflammation, and oxidative stress [19]. These findings demonstrate the therapeutic potential of DHEA in various inflammatory conditions. However, the role of DHEA and DHEAS in AP remains unclear. We speculate that lower levels of DHEAS in BALF, serum, and sputum may indicate a diminished anti-inflammatory response, potentially exacerbating lung injury and the inflammatory cascade in AP. Supplementing DHEAS may modulate this response and improve outcomes in AP. Future research is needed to elucidate the specific role of DHEAS in AP progression. As an ideal diagnostic marker, DHEAS should not only exhibit good sensitivity and specificity but also stable performance and ease of detection. DHEAS serum levels have established reference ranges in various age groups [15], and besides LC-MS/MS analysis, methods such as enzyme-linked immunosorbent assay (ELISA) and radioimmunoassay are currently used for its detection. Given its clinical applicability, DHEAS could serve as a promising biomarker for AP.

ADIOLS, the sulfate form of androst-5-ene-3beta,17beta-diol (ADIOL) [20], is a downstream metabolite of DHEA [21]. ADIOL has been recognized for its antioxidant, anti-inflammatory, and anti-apoptotic effects [22]. Studies have demonstrated that ADIOL exerts protective effects in models of colitis and sepsis [23, 24]. Notably, in a study of Enterococcus faecalis-induced sepsis, 100% of ADIOL-treated mice survived compared to 57% survival in untreated mice. In our study, ADIOLS levels were significantly decreased in AP patients, suggesting that ADIOL may play a protective role in lung inflammation. Given its potential to modulate inflammatory responses and reduce pro-inflammatory cytokine release, ADIOL could be explored as an adjuvant therapy for AP, alongside conventional treatments, to reduce inflammation and prevent recurrence.

Tauro-beta-muricholic acid, the conjugated form of beta-muricholic acid, is a potent Farnesoid X receptor (FXR) antagonist that regulates bile acid metabolism, innate immunity, and inflammatory responses [25, 26]. FXR downregulates enzymes such as cholesterol 7α-hydroxylase, sterol 12D-hydroxylase and sterol 27-hydroxylase through a negative feedback mechanism [27]. Recent studies have also highlighted bile acids as key signaling molecules in the regulation of the immune system [14]. In our study, we observed a significant increase in beta-muricholic acid in BALF, sputum, and serum samples from AP patients. We hypothesize that beta-muricholic acid may influence immune responses, inflammatory processes, and bile acid metabolism in AP by inhibiting FXR. Disruption of FXR signaling and bile acid homeostasis may contribute to the inflammatory pathways observed in AP, suggesting that beta-muricholic acid could serve as a potential therapeutic target for intervening in these inflammatory processes.

This study included 9 AP cases requiring ICU admission, revealing a significant 8:1 male-to-female ratio. This gender imbalance may reflect the higher incidence of AP in males, who are more likely to progress to critical stages. Previous studies have established that male sex is a risk factor for AP [28, 29], with males being more prone to severe outcomes. An animal study also demonstrated sex-based differences in AP severity, with males exhibiting greater vulnerability to lung injury [30]. Additionally, DHEAS, a selected differential metabolite, can be converted into estrogen in the lungs [31]. This gender difference may be related to the protective effects of estrogen.

While these findings provide valuable insights, several limitations should be considered. First, the relatively small sample size (*n* = 19) limits the generalizability of our results. Second, the use of sedatives and corticosteroids in some patients may have influenced the metabolite levels observed in this study. Future research should account for these potential confounders and validate the findings in larger, independent cohorts to assess the robustness of the identified biomarkers.

Finally, while our study highlights promising biomarkers for AP diagnosis, further exploration of their functional roles in AP pathogenesis is essential. Experimental studies using cell culture models and AP animal models could provide insights into how DHEAS, ADIOLS, and beta-muricholic acid modulate immune responses and inflammatory processes in AP. Longitudinal studies could also assess whether these biomarkers correlate with disease progression and response to treatment.

Future directions should focus on validating these biomarkers in larger cohorts and investigating their mechanistic roles in AP pathophysiology. These efforts could pave the way for the development of diagnostic tools and targeted therapies for AP.

## Conclusions

This study identifies DHEAS, ADIOLS, and beta-muricholic acid as promising biomarkers for AP. These metabolites show significant potential for improving early diagnosis and guiding treatment strategies. Our findings underscore the clinical value of metabolomics in developing diagnostic tools for AP, which could lead to better clinical management and improved patient outcomes.

## Electronic supplementary material

Below is the link to the electronic supplementary material.

Supplementary Material 1: Supplementary Methods. Table S1 Number of missing values and corresponding dispositions. Fig. S1 QCs RSD Coverage. Fig. S2 Analysis of the abundance of selected molecules by Box plots. Fig. S3 KEGG pathway enrichment analysis of metabolites with significant differences in different samples.

Supplementary Material 2: Table S2 Metabolites identified in bronchoalveolar lavage fluid. Table S3 Metabolites identified in saliva. Table S4 Metabolites identified in serum. Table S5 Metabolites identified in sputum. Table S6 Metabolites identified in urine.

Supplementary Material 3: Table S7 Significantly differential metabolites identified in bronchoalveolar lavage fluid. Table S8 Significantly differential metabolites identified in saliva. Table S9 Significantly differential metabolites identified in serum. Table S10 Significantly differential metabolites identified in sputum. Table S11 Significantly differential metabolites identified in urine.

Supplementary Material 4: Table S12 Enrichment analysis of metabolites dysregulated in bronchoalveolar lavage fluid. Table S13 Enrichment analysis of metabolites dysregulated in serum. Table S14 Enrichment analysis of metabolites dysregulated in sputum. Table S15 Enrichment analysis of dysregulated metabolites in all samples.

Supplementary Material 5: Table S16. The resulting correlation matrix.

## Data Availability

The datasets used and/or analysed during the current study are available from the corresponding author on reasonable request.

## Abbreviations

AP: Aspiration pneumonia
BALF: Bronchoalveolar lavage fluid
LC-MS/MS: Liquid chromatography with tandem mass spectrometry
AUC: Area under curve
DHEAS: Dehydroepiandrosterone sulfate
ADIOLS: Androstenediol-3-sulfate
APACHE II: Acute Physiology and Chronic Health Evaluation II
VFSS: Videofluoroscopic swallowing study
FEES: Flexible endoscopic evaluation of swallowing
ICU: Intensive care unit
QC: Quality Control
IQR: Interquartile range
SD: Standard deviation
KEGG: Kyoto encyclopedia gene and genome database
ROC: Receiver operating characteristic
RSD: Relative standard derivation
DHEA: Dehydroepiandrosterone
ADIOL: Androst-5-ene-3beta,17beta-diol
FXR: Farnesoid X receptor
DDA: Data-dependent acquisition
RT: Retention time
KNN: K-Nearest Neighbors
VSN: Variance stabilizing normalization
PLS-DA: Partial least squares discriminant analysis
