# Supplementary material for: Study on the potential diagnostic value of metabolomics changes in different biological fluids for aspiration pneumonia
Source: BMC Pulm Med. 2025 Feb 4;25:60. doi: 10.1186/s12890-025-03519-x (PMC11792630; doi:10.1186/s12890-025-03519-x)
Supplement: Supplementary file 1 — Supplementary Material 1: Supplementary Methods. Table S1 Number of missing values and corresponding dispositions. Fig. S1 QCs RSD Coverage. Fig. S2 Analysis of the abundance of selected molecules by Box plots. Fig. S3 KEGG pathway enrichment analysis of metabolites with significant differences in different samples. [file 12890_2025_3519_MOESM1_ESM.docx]

**Additional file1**

**Supplementary Methods**

**LC-MS/MS analysis.** DDA metabolomics analysis was performed using a Thermo Vanquish (Thermo Fisher Scientific) with Q Exative Plus orbitrap high resolution mass spectrometry (Thermo Fisher Scientific, USA), data acquisition software XCalibur 4.3 (Thermo Fisher Scientific). All QC samples, blank samples, and experimental samples were randomized, and QC samples spaced evenly among the injections. In this experiment, a total of three QC injections were analyzed. The main parameters are as follows. C18 detection mode: mobile phase was water/methanol, solvent was added with 0.1% FA and 5 mM ammonium formate, AcquityTM BEH C18 column (Waters Co., USA, 1.7 μm, 2.1×100 mm), separation gradient: the organic phase was increased from 2% to 100% in 12 min, and the remaining 6 min was used to flush and equilibrate the column; flow rate: 0.3 mL/min, injection volume: 5 μL, column temperature: 40 °C. The detection was carried out in positive and negative ion modes, respectively.

HILIC detection mode: mobile phase was water/acetonitrile, solvent was added with 25 mM ammonium formate buffer and adjusted to pH 9.0, Acquity^TM^ BEH AMIDE column (Waters Co., USA, 1.7 μm, 2.1×100 mm), separation gradient: the organic phase increased from 2% to 98% in 10 min, and the extra 2 min was used to wash and equilibrate the column; flow rate: 0.4 mL/min, column temperature: 50 °C. The detection was carried out in positive and negative ion modes, respectively.

Mass spectrometer: spray voltage: 4.0 kV(+) and 3.5 KV(-); capillary temperature: 320 °C; S-Lens: 55%; collision energy: 20、40、60% HCD; sheath gas flow; 45 arb, auxiliary gas flow rate: 10 arb, resolution setting: first level 70,000@m/z 200, two Level 17,500@m/z 200; Max IT: full MS 200 ms, full MS/MS 45 ms; parent ion scanning range: m/z 70-1050, dynamic exclusion 40 s.

**Table S1** Number of missing values and corresponding dispositions

|  | No. of missing value | Disposition |
| --- | --- | --- |
| WBC | 1(5.3%) | multiple imputation |
| PCT | 1(5.3%) | multiple imputation |
| Cholesterol | 2(10.5%) | multiple imputation |
| Albumin | 1(5.3%) | multiple imputation |
| D-BIL | 2(10.5%) | multiple imputation |
| I-BIL | 2(10.5%) | multiple imputation |
| Lac | 6(31.6) | multiple imputation |
| APACHE II score | 7(36.8%) | multiple imputation |
| Corticosteroids | 1(5.3%) | multiple imputation |
| Hospital LOS | 1(5.3%) | multiple imputation |

WBC: white blood cell; PCT: procalcitonin; D-BIL: direct bilirubin; I-BIL: indirect bilirubin; Lac: lactic acid; APACHE II: Acute Physiology and Chronic Health Evaluation II; LOS: length of stay

**
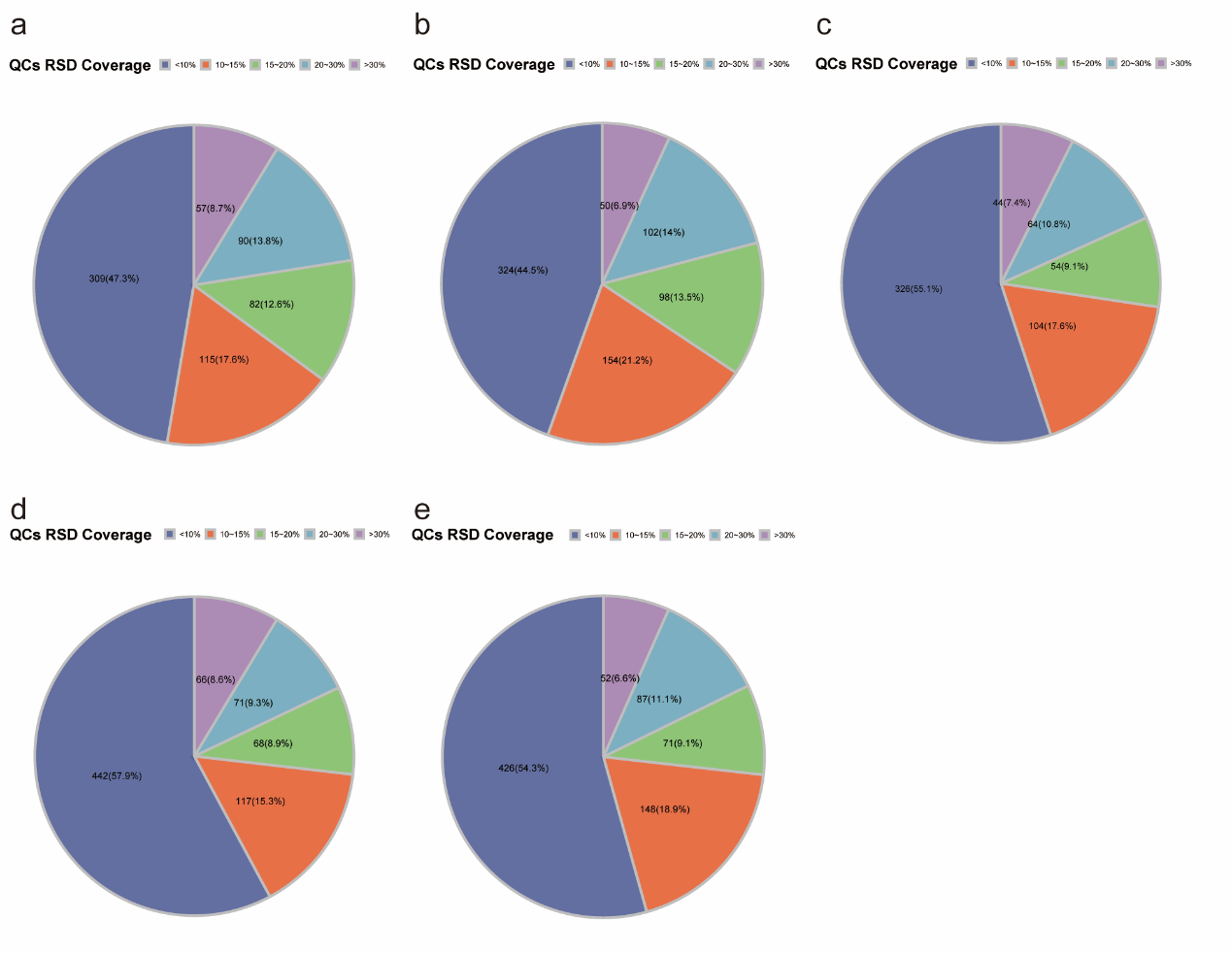
**

**Fig. S1 QC RSD Coverage.** (a) Relative Standard Deviation (RSD) coverage in QC BALF samples (596 metabolites passing quality control). RSD is calculated as the ratio of the mean to the standard deviation for each metabolite. Metabolites are classified based on RSD values: Blue (<10%), Red (10%-15%), Green (15%-20%), Light Blue (20%-30%), and Purple (>30%). (b-e) RSD coverage for QC saliva (678 metabolites passing quality control), serum (548 metabolites passing quality control), sputum (698 metabolites passing quality control), and urine (732 metabolites passing quality control) samples, with the same color coding applied to categorize metabolites based on RSD values.

**
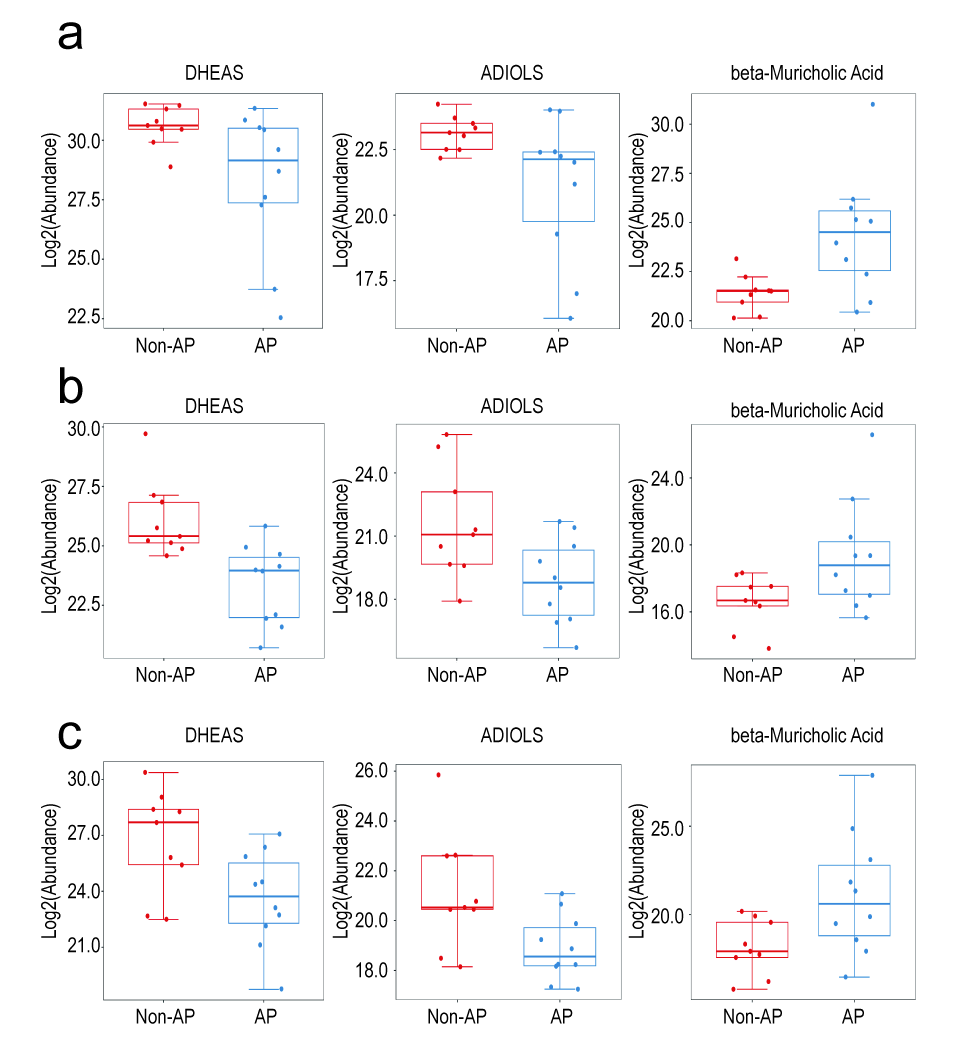
**

**Fig. S2 Analysis of the abundance of selected molecules by Box plots.** (a-c) Box plots show the abundance of selected molecules in serum, bronchoalveolar lavage fluid (BALF), and sputum samples, respectively. The Y axis represents the log2-transformed abundance, and the X axis represents the grouping.


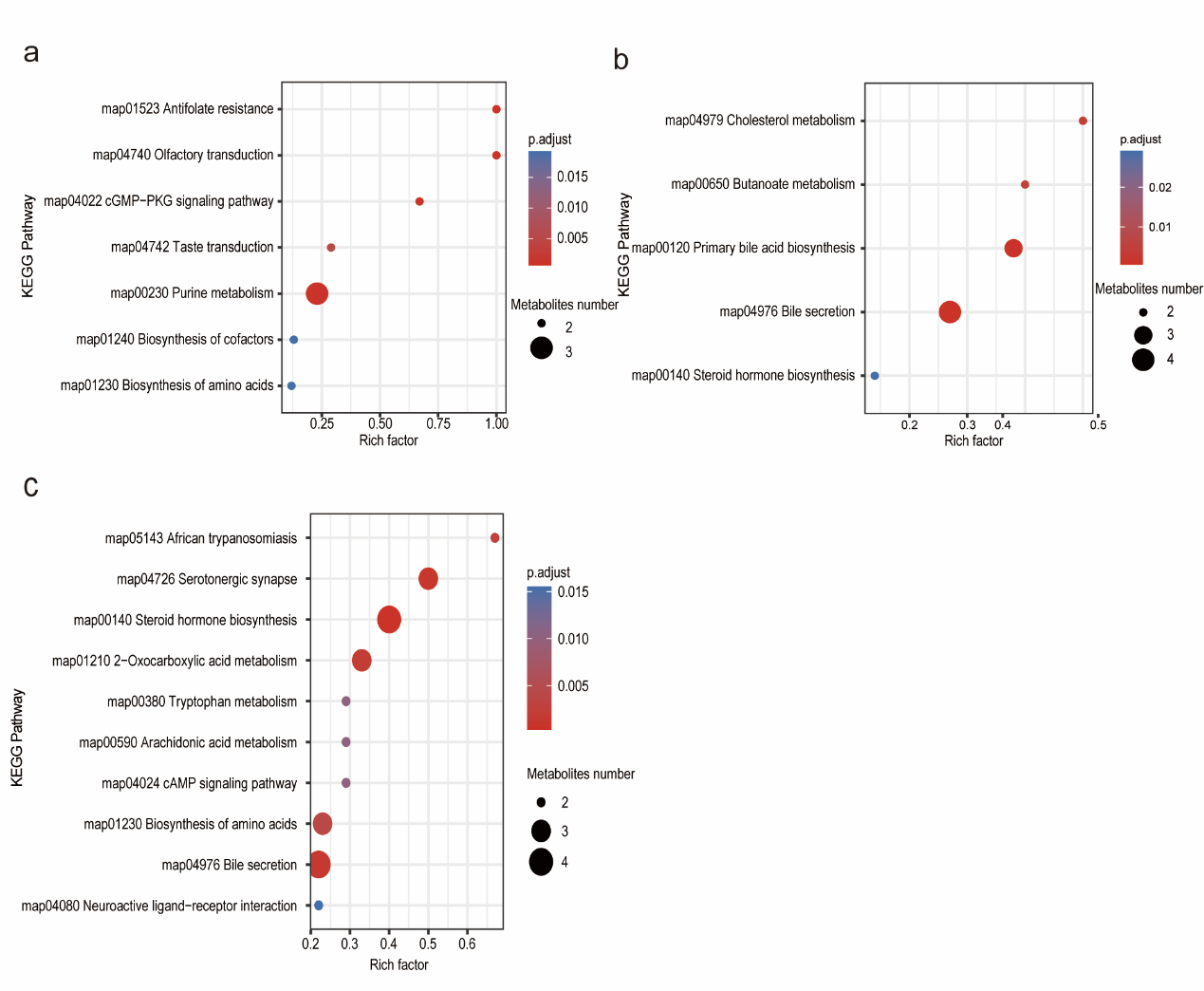


**Fig. S3 KEGG pathway enrichment analysis of metabolites with significant differences in different samples.** (a) KEGG pathway enrichment analysis of metabolites with significant differences in bronchoalveolar lavage fluid (BALF) samples. The Y axis represents the significantly enriched KEGG pathways, and the X axis represents the proportion of differential metabolites in each KEGG pathway. The color of the bubbles indicates the significance of the enriched KEGG pathways (P value), with color changes reflecting the variation in P value. The size of each bubble corresponds to the number of differential metabolites involved in the KEGG pathway. (b-c) KEGG pathway enrichment analysis of metabolites with significant differences in serum and sputum samples.
