# Supplementary material for: Study on the potential diagnostic value of metabolomics changes in different biological fluids for aspiration pneumonia
Source: BMC Pulm Med. 2025 Feb 4;25:60. doi: 10.1186/s12890-025-03519-x (PMC11792630; doi:10.1186/s12890-025-03519-x)
Supplement: Supplementary file 5 — Supplementary Material 5: Table S16. The resulting correlation matrix. [file 12890_2025_3519_MOESM5_ESM.docx]

**Additional file5**

**Table S16** The obtained correlation matrix

| Row data | Col data | *r* | *P* value | Relation |
| --- | --- | --- | --- | --- |
| BALF_DHEAS | APACHE | -0.619 | 0.005 | negative |
| Serum_beta-Muricholic Acid | WBC | 0.449 | 0.054 | positive |
| Sputum_DHEAS | APACHE | -0.438 | 0.061 | negative |
| BALF_ADIOLS | APACHE | -0.428 | 0.068 | negative |
| Serum_DHEAS | APACHE | -0.392 | 0.097 | negative |
| Serum_beta-Muricholic Acid | APACHE | 0.363 | 0.127 | positive |
| Serum_ADIOLS | APACHE | -0.358 | 0.133 | negative |
| Sputum_ADIOLS | APACHE | -0.343 | 0.150 | negative |
| Sputum_ADIOLS | PCT | 0.342 | 0.151 | positive |
| BALF_beta-Muricholic Acid | APACHE | 0.330 | 0.168 | positive |
| Serum_ADIOLS | PCT | 0.312 | 0.194 | positive |
| Sputum_beta-Muricholic Acid | APACHE | 0.296 | 0.218 | positive |
| Sputum_beta-Muricholic Acid | WBC | 0.273 | 0.258 | positive |
| BALF_beta-Muricholic Acid | WBC | 0.264 | 0.274 | positive |
| BALF_DHEAS | PCT | 0.213 | 0.381 | positive |
| Serum_beta-Muricholic Acid | PCT | -0.200 | 0.411 | negative |
| Sputum_beta-Muricholic Acid | PCT | 0.189 | 0.439 | positive |
| Sputum_DHEAS | PCT | 0.152 | 0.535 | positive |
| Serum_DHEAS | WBC | -0.112 | 0.647 | negative |
| Sputum_DHEAS | WBC | 0.090 | 0.713 | positive |
| BALF_ADIOLS | PCT | 0.090 | 0.716 | positive |
| BALF_ADIOLS | WBC | 0.084 | 0.732 | positive |
| BALF_beta-Muricholic Acid | PCT | 0.075 | 0.762 | positive |
| Serum_DHEAS | PCT | -0.053 | 0.830 | negative |
| Serum_ADIOLS | WBC | 0.050 | 0.839 | positive |
| Sputum_ADIOLS | WBC | -0.047 | 0.847 | negative |
| BALF_DHEAS | WBC | -0.031 | 0.901 | negative |

BALF: bronchoalveolar lavage fluid; DHEAS: dehydroepiandrosterone sulfate; ADIOLS: androstenediol-3-sulfate; WBC: white blood cell; PCT: procalcitonin; APACHE II: Acute Physiology and Chronic Health Evaluation II
